# Widespread Increase of Functional Connectivity in Parkinson’s Disease with Tremor: A Resting-State fMRI Study

**DOI:** 10.3389/fnagi.2015.00006

**Published:** 2015-02-03

**Authors:** Delong Zhang, Xian Liu, Jun Chen, Bo Liu, Jinhui Wang

**Affiliations:** ^1^Department of Radiology, Guangdong Provincial Hospital of Chinese Medicine, Guangzhou, China; ^2^Guangzhou University of Chinese Medicine Postdoctoral Mobile Research Station, Guangzhou, China; ^3^Center for Cognition and Brain Disorders, Hangzhou Normal University, Hangzhou, China; ^4^Zhejiang Key Laboratory for Research in Assessment of Cognitive Impairments, Hangzhou, China

**Keywords:** Parkinson’s disease, tremor, connectome, centrality, resting functional connectivity

## Abstract

Parkinson’s disease (PD) is a clinically heterogeneous disease in the symptomatology dominated by tremor, akinesia, or rigidity. Focusing on PD patients with tremor, this study investigated their discoordination patterns of spontaneous brain activity by combining voxel-wise centrality, seed-based functional connectivity, and network efficiency methods. Sixteen patients and 20 matched healthy controls (HCs) were recruited and underwent structural and resting-state functional MRI scan. Compared with the HCs, the patients exhibited increased centrality in the frontal, parietal, and occipital regions while decreased centrality in the cerebellum anterior lobe and thalamus. Seeded at these regions, a distributed network was further identified that encompassed cortical (default mode network, sensorimotor cortex, prefrontal and occipital areas) and subcortical (thalamus and basal ganglia) regions and the cerebellum and brainstem. Graph-based analyses of this network revealed increased information transformation efficiency in the patients. Moreover, the identified network correlated with clinical manifestations in the patients and could distinguish the patients from HCs. Morphometric analyses revealed decreased gray matter volume in multiple regions that largely accounted for the observed functional abnormalities. Together, these findings provide a comprehensive view of network disorganization in PD with tremor and have important implications for understanding neural substrates underlying this specific type of PD.

## Introduction

Parkinson’s disease (PD) is a neurodegenerative disorder typically characterized by motor symptoms, but distinct symptoms also include tremor, akinesia, and rigidity (Deuschl et al., 2000; Lees et al., 2009). Such a symptomatic heterogeneity is a key confounding factor in disclosing the pathophysiology of PD (Jankovic et al., 1990). Uncovering neural substrates accounting for this heterogeneity is critically important to advance our knowledge of PD and discover efficient therapies.

Pathophysiologically, PD is commonly thought to be attributed to the dysfunction of the basal ganglia circuit (i.e., the striatal-thalamic-corticoloop) triggered by deficits in dopaminergic nigrostriatal neurons (Hacker et al., 2012). However, despite success in accounting for the parkinsonian symptoms of akinesia and rigidity, this theory fails to explain tremor (Helmich et al., 2011). In clinical studies, the level of tremor severity is independent of the amount of dopamine deficiency (Toth et al., 2004; Lees, 2007; Helmich et al., 2012) and is often insensitive to dopamine treatment (Fishman, 2008; Rodriguez-Oroz et al., 2009). This is consistent with the findings from post-mortem studies, which show that PD patients with tremor have less dopaminergic dysfunction than non-tremor PD patients (Paulus and Jellinger, 1991; Jellinger, 1999). These findings suggest that PD with tremor is a unique and characteristic disease state in PD. However, brain mechanism underlying such specific state of PD is not well-established.

Recently, neuroimaging techniques have been used to reveal structural and functional brain alterations in PD patients with tremor. Compared with healthy controls (HCs), PD patients with tremor are found to show increased gray matter (GM) concentration (Kassubek et al., 2002) and hypermetabolism (Kassubek et al., 2001) in the thalamus, increased neural activity in the dorsolateral prefrontal cortex (Prodoehl et al., 2013), and elevated functional connectivity between the basal ganglia and the cerebello-thalamo-cortical circuit (Helmich et al., 2012). These studies suggest a wide involvement of multiple sites ranging from cortical to subcortical regions in PD with tremor, therefore triggering a hypothesis that there may exist a distributed network associated with PD with tremor. Given the interconnected nature of the brain to integrate various information inputs across segregated sensory systems (Bullmore and Sporns, 2009; He and Evans, 2010; Van Dijk et al., 2010), such a network hypothesis is reasonable but still lacks direct support from empirical evidence.

To fully depict abnormal brain networks in PD patients with tremor, the current study combined several approaches and applied them to resting-state functional MRI (R-fMRI) data collected from 16 PD patients with tremor and 20 matched HCs. R-fMRI measures spontaneous brain activity (Biswal et al., 1995) and is proposed as a promising tool to map intrinsic brain networks (Van Dijk et al., 2010). We first constructed individual whole-brain, voxel-level functional connectivity networks. A graph-based measure, degree centrality, was then employed to locate the brain sites exhibiting abnormal functional connectivities in the patients. The measure is independent of the prior definition of regions of interest (ROIs), therefore providing an unbiased approach to test functional discoordination by searching over the entire brain. The identified sites were subsequently used as seed regions to trace the brain regions to which the abnormal functional connectivities were linked, therefore outlining the whole discoordination landscape in PD with tremor. Furthermore, we performed a network efficiency analysis to characterize the topological organization of the abnormal connectivity network identified in the patients. Finally, we correlated the disorganized network to clinical variables of the patients and tested their potential to serve as biomarkers in discriminating the disease. In addition, we also examined regional GM volume changes in the patients and tested the extent to which structural alterations contributed to functional abnormalities.

## Materials and Methods

### Participants

Thirty-six right-handed participants, comprising 16 PD patients with tremor (9 men and 7 women) and 20 age-, gender-, and education-matched HCs (11 men and 9 women) were recruited from the Second Affiliated Hospital of Guangzhou University of Traditional Chinese Medicine (Guangdong Province’s Traditional Chinese Medical Hospital) in the present study. All the patients underwent a detailed clinical assessment of history of family genetic and traumatic brain injuries, neurological examinations, including the Unified Parkinson’s Disease Rating Scale (UPDRS), Hoehn and Yahr Scale (H–Y stage), and a conventional MRI scan. All the clinical assessments and MRI scans were performed when the patients were in their off-medicine condition (i.e., at least 8–12 h after the last dose of dopaminergic medication) to avoid a medication effect as much as possible. The patients were diagnosed by an experienced neurologist (XL) according to the UK PD Brain Bank Criteria (Gibb and Lees, 1988). Inclusion criterion was the presence of the resting tremor in the unilateral or bilateral upper or lower extremity. All the selected PD patients had classical parkinsonian resting tremor with (*n* = 14) or without (*n* = 2) action or postural tremor. There were no cognitive impairments for each individual PD patient, as measured by Mini-Mental State Examination (>28, mean = 29.8 ± 0.05). The participants were excluded for advanced PD stages (H–Y ≥ 4), secondary parkinsonism, atypical parkinsonian disease, and a history of any substance dependence, head trauma, or claustrophobia. All the participants gave written informed consent for the present study. This study was approved by the Institutional Review Board of the Guangzhou University of Traditional Chinese Medicine. Table 1 lists detailed demographic and clinical information for all the participants.

**Table 1:** **Demographics and clinical characteristics of the participants**.

	HC (*n* = 20)	PD (*n* = 16)	*p* value
Age (yrs)	42–78 (59.2 ± 8.7)	37–81 (60.5 ± 11.8)	0.37^b^
Gender (M/F)	11/9	9/7	0.90^a^
Education (yrs)	0–22 (11.4 ± 5.0)	0–20 (9.8 ± 4.2)	0.14^b^
Illness duration (yrs)	–	0.42–6 (2.5 ± 1.7)	–
MMSE	–	29.0–30 (29.8 ± 0.05)	–
UPDRS	–	4–49 (27.3 ± 14.3)	–
H–Y	–	1–3 (2.25 ± 0.91)	–
Tremor level	–	1–4 (2 ± 0.85)	–

*Data are presented as minimum–maximum (Mean ± SD). PD, Parkinson’s disease; HC, healthy control; MMSE, Mini-Mental State Examination; UPDRS, Unified Parkinson’s Disease Rating Scale; H–Y, Hoehn and Yahr Scale*.

*^a^The *p* value was obtained using a two-tail Pearson chi-square test*.

*^b^The *p* values were obtained using two-sample two-tail *t*-tests*.

### Image acquisition

All the participants were scanned using a 1.5 T MR scanner (Siemens Magnetom Avanto, Erlangen, Germany) at the department of radiology of the Second Affiliated Hospital of Guangzhou University of Traditional Chinese Medicine. R-fMRI data were collected using an echo-planar imaging sequence: 30 axial slices; repetition time (TR) = 2000 ms; echo time (TE) = 39 ms; slice thickness = 4 mm; gap = 1 mm; flip angle (FA) = 90°; matrix = 64 × 64; field of view (FOV) = 240 mm × 240 mm. During the data acquisition, the participants were asked to lie quietly in the scanner with their eyes closed. After scanning, a total of 180 volumes were obtained for each participant. Individual high-resolution 3D structural images were also acquired using a T1-weighted MP-RAGE sequence: 192 axial slices; TR = 1160 ms; TE = 4.21 ms; inversion time = 600 ms; slice thickness = 0.9 mm; no gap; FA = 15°; matrix = 512 × 512; FOV = 256 mm × 256 mm.

### Data preprocessing

Resting-state functional MRI data preprocessing was performed with the GRETNA toolbox^1^ based on SPM8^2^. After removal of the first five volumes, the functional images were corrected for time offsets between slices and geometrical displacements due to head movement. None of the participants were excluded based on the criterion of a displacement of >3 mm or an angular rotation of >3° in any direction. The summary scalars of both gross (maximum and root mean square) and micro (mean frame-wise displacement) head motions were matched between the PD patients and HCs (all *Ps* > 0.15). All the corrected functional data were then normalized to the Montreal Neurological Institute space using an optimum 12-parameter affine transformation and non-linear deformations and then resampled to a 3-mm isotropic resolution. The resulting images were further temporally band-pass filtered (0.01–0.1 Hz) to reduce the effects of low-frequency drift and high-frequency physiological noise, and linear trend was also removed. Finally, several nuisance signals were regressed out from each voxel’s time course, including 24-parameter head-motion profiles (Friston et al., 1996; Yan et al., 2013), mean white matter (WM), and cerebrospinal fluid (CSF) time series within the respective brain masks derived from prior probability maps in SPM8 (threshold = 0.8). Of note, spatial smoothing was not included in the data preprocessing as in previous studies (Zuo et al., 2012) since smoothing could induce spurious local correlations for subsequent centrality analysis.

For structural images, we performed a voxel-based morphometry (VBM) analysis to determine GM volume alterations in the patients and to examine the potential effect of structural changes on functional abnormalities. Briefly, individual GM volume maps were obtained through the following steps: (i) segmentation of individual structural images into GM, WM, and CSF based on an adaptive Maximum A Posterior technique; (ii) normalization of the resulting GM maps into the Montreal Neurological Institute space using a high-dimensional DARTEL approach; (iii) non-linear modulation of GM maps to compensate for spatial normalization effects; and (iv) spatial smoothing of GM maps using a 6-mm full width at the half-maximum Gaussian kernel. Notably, the non-linear modulation essentially corrected for individual brain sizes. Structural data preprocessing was performed with the VBM8 toolbox for SPM8^3^.

### Voxel-wise weighted degree centrality

Weighted degree centrality (WDC) is a measure from graph theory that quantifies the importance/centrality of a node in a network in terms of its connectivity strength to all the other nodes. For the voxel-wise centrality analysis, a node represents a voxel and the inter-node connectivity strength represents inter-voxel functional connectivity in their BOLD signals. Formally, the WDC for a given voxel *i* is calculated as follows:

(1) $$S\left(i\right)=\sum_{j=1}^{N}r_{\mathrm{ij}}-1$$

where *r_ij_* is the Pearson correlation coefficient between voxel *i* and voxel *j* in their BOLD signal time series and *N* is the number of GM voxels according to the GM probability map in SPM8 (threshold = 0.2). To avoid the contamination of spurious weak correlations, a threshold is necessary before the summation. In contrast to an arbitrary choice, we employed a thresholding procedure based on the statistical significance level of the correlation analyses. Specifically, correlations surviving at a threshold of *P* < 0.05 (Bonferroni corrected) were screened and retained or were reset to 0. Of note, during the calculation, negative correlations were excluded given their ambiguous interpretation and detrimental effects on test–retest reliability (Fox et al., 2009; Murphy et al., 2009; Weissenbacher et al., 2009; Wang et al., 2011). After calculating WDC for each voxel, a whole-brain map was obtained for each participant, with the value at a given voxel indicating its functional integration over the entire brain. Degree centrality has been widely used in brain network studies (Buckner et al., 2009; Tomasi and Volkow, 2010; Zuo et al., 2012; Di Martino et al., 2013) due to its simplicity in understanding and implementation and high test–retest reliability (Wang et al., 2011; Cao et al., 2014).

### Seed-based functional connectivity

Although whole-brain voxel-wise WDC analysis allows us to identify the brain sites that exhibit abnormal functional connectivity between PD patients and HCs (seven regions were identified in this study), we did not obtain any insight regarding the locations to which these abnormal connections were linked. To trace these locations, we further performed a seed-based functional connectivity analysis. Specifically, a sphere ROI was generated for each of the seven regions, with the centroid at the corresponding peak voxel (radius = 6 mm); the mean time series was then extracted and correlated to all the other voxels in the brain. This resulted in seven correlation maps for each participant, which further underwent Fisher’s *r*-to-*z* transformation to improve the normality.

### Network efficiency

The voxel-wise WDC and subsequent seed-based functional connectivity analyses identified a total of 57 regions that exhibited abnormal functional connectivity in PD patients with tremor. To uncover the organization among these regions, we further constructed their pairwise connectivity matrix individually and fed them into graph-based analyses. Briefly, a sphere ROI was generated for each of the 57 regions, with the centroid at the corresponding peak voxel (radius = 6 mm). Seven ROIs were excluded to avoid spatial overlapping. For each of the remaining 50 ROIs, a mean time series was extracted for each participant and then correlated with all the other ROIs, therefore outputting a 50 × 50 correlation matrix. To exclude possible effects of spurious correlations on network topology, a sparsity threshold (i.e., the ratio of the number of existing edges divided by the maximum possible number of edges in a network) was applied to individual correlation matrices employed to convert individual correlation matrices such that only those high correlations are remained. The sparsity approach normalized all resultant networks to have the same number of nodes and edges and minimized the effects of discrepancies in the overall correlation strength between groups. However, because there is currently no definitive way to select a single threshold, we therefore empirically thresholded each correlation matrix repeatedly over a wide range of 0.08 ≤ sparsity ≤ 0.6 (interval = 0.02) to obtain sparse and weighted networks. For the resultant networks at each sparsity, we calculated global and local efficiency to characterize parallel information flow within them (Latora and Marchiori, 2003; Achard and Bullmore, 2007). Similar to previous studies (He et al., 2009; Zhang et al., 2011a), we also calculated the area under the curve (AUC) for each network metric (global and local efficiency) to provide a summarized scalar independent of single threshold selection. Mathematically, the global efficiency for a network *G* is defined as:

(2) $$E_{\mathrm{glob}}\left(G\right)=\frac{1}{N\left(N-1\right)}\sum_{i\neq j\in G}\frac{1}{d_{\mathrm{ij}}}$$

where *d_ij_* is the shortest path length between node *i* and node *j* in *G* and is calculated as the smallest sum of edge lengths throughout all of the possible paths from node *i* and node *j*. The length of an edge was designated as the reciprocal of the edge weight (i.e., correlation coefficient), which can be interpreted as a functional distance that a high correlation coefficient corresponds to a short functional distance. Global efficiency measures the ability of parallel information transmission over the network. The local efficiency of *G* is measured as:

(3) $$E_{\mathrm{loc}}\left(G\right)=\frac{1}{N}\sum_{i\in G}E_{\mathrm{glob}}\left(G_{i}\right)$$

where *E_glob_*(*G_i_*) is the global efficiency of *G_i_*, the subgraph comprised the neighbors of the node *i* (i.e., nodes linked directly to node *i*). Local efficiency measures the fault tolerance of the network, indicating the capability of information exchange for each subgraph when the index node is eliminated.

To determine whether the constructed brain networks were topologically organized into small-world architectures, the global and local efficiency were normalized by the corresponding mean derived from 100 random networks that preserved the same number of nodes, edges, and degree distributions as the real brain networks (Maslov and Sneppen, 2002; Milo et al., 2002). Typically, a network is thought to be small-world if it has a normalized local efficiency larger than 1 and a normalized global efficiency approximately equal to 1.

### Statistical analysis

#### Between-group comparison

Two-sample *t*-tests were used to determine the between-group differences in GM volume, WDC, and seed-based functional connectivity. Non-parametric permutation tests (10,000 permutations) were used to test between-group differences in network measure (global and local efficiency). Gender and age were treated as unconcerned covariates for all these comparisons. Summary head-motion variables (maximum, root mean square, and mean frame-wise displacement) were treated as extra covariates for all functional comparisons (Fair et al., 2012). Additionally, voxel-specific GM volumes were added as covariates to test the structural contribution to functional abnormalities in WDC. For volume-based comparisons (GM volume, WDC, and seed-based functional connectivity), the results are presented at a statistical threshold of *P* < 0.05 (corrected) by combining a height threshold and an extent threshold determined by Monte Carlo simulations (Ledberg et al., 1998). For graph-based metrics, a false discovery rate (FDR) procedure was used to correct for multiple comparisons across different sparsities.

#### Brain–behavior correlation

Multiple partial Spearman correlation analyses were performed to examine the relationship between the neuroimaging results (WDC, functional connectivity, network efficiency, and GM volume) and clinical variables (duration, tremor, H–Y scale, and UPDRS score) for the patients after controlling for the corresponding confounding mentioned above.

### Classification

To determine whether the observed between-group differences could be used to discriminate the patients from HCs, we implemented a receiver operating characteristic curve (ROC) analysis with the public code^4^ (Giuseppe Cardillo, Naples, Italy).

## Results

### Demographic and clinical characteristics

Table 1 summarizes the detailed demographic and clinical characteristics of all the participants. There were no significant between-group differences in age, gender, or years of education (all *Ps* > 0.37). The tremor score (an item in the UPDRS), UPDRS, H–Y, and disease duration of the patients were 2.0 ± 0.85, 27.3 ± 14.3, 2.25 ± 0.91, and 2.5 ± 1.7 years, respectively.

### Altered degree centrality in PD with tremor

**Figure 1:**
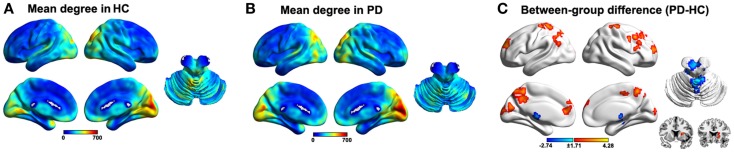
**Results of the within/between-group analysis on weighted degree centrality (WDC)**. **(A)**, Mean WDC pattern for the HC group. **(B)**, Mean WDC pattern for the PD group. **(C)**, Between-group differences in the WDC. The results were mapped onto the brain surface using the BrainNet viewer (http://www.nitrc.org/projects/bnv/).

We utilized an unbiased voxel-wise WDC approach to explore abnormal functional connectivity networks in PD patients with tremor. The mean WDC distributed heterogeneously across the entire brain, with the most highly connected regions predominantly in the posterior parietal and occipital, lateral temporal, and medial prefrontal cortices and the cerebellum, a common pattern in both the HC (Figure 1A) and PD (Figure 1B) groups. Nevertheless, between-group comparison revealed widely altered WDC in the PD group (Figure 1C; Table S1 in Supplementary Material). Specifically, six clusters were observed to show increased WDC in the PD group, predominantly involving in the prefrontal (superior/middle/inferior frontal gyri and precentral gyrus), parietal (postcentral gyrus, precuneus, and superior parietal lobule), and occipital (calcarine and cuneus) regions. Notably, portions of the putamen and caudate also exhibited increased WDC and are thought to play critical roles in PD pathology. With regard to decreased WDC, only one cluster was detected and primarily encompassed the left cerebellum anterior lobe and bilateral thalamus.

### Altered functional connectivity in PD with tremor

**Figure 2:**
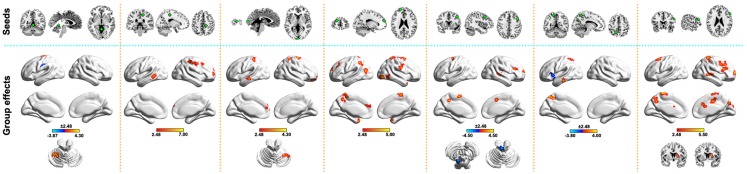
**Between-group differences in seed-based functional connectivity**. The seeds (top row) were defined as spherical ROIs (radius = 6 mm) centered at the peak voxels, with the strongest group effects in WDC for clusters in Figure 1C. Regions showing abnormal functional connectivity in the PD (bottom row) were mapped onto the brain surface using the BrainNet viewer (http://www.nitrc.org/projects/bnv/). See Table S1 in Supplementary Material for detailed information.

To locate sites to which the above-mentioned regions were abnormally linked, we further performed a seed-based functional connectivity analysis. In total, 50 brain regions were identified as showing abnormal functional connectivity to the seven above-mentioned clusters in the PD patients (Figure 2). These regions distributed predominantly within the DMN (e.g., left precuneus, inferior parietal lobule, and middle/inferior temporal gyri), sensorimotor (e.g., left postcentral gyrus, supplementary motor area, paracentral lobule, and right precentral gyrus), and prefrontal [e.g., right inferior/middle frontal gyri and left superior frontal gyrus (SFG)] cortices. Notably, all the altered functional connectivities were increased in the PD group compared with the HC group, except for four connections. Table S1 in Supplementary Material lists the detailed information of these abnormal connectivities.

### Altered network efficiency in PD with tremor

**Figure 3:**
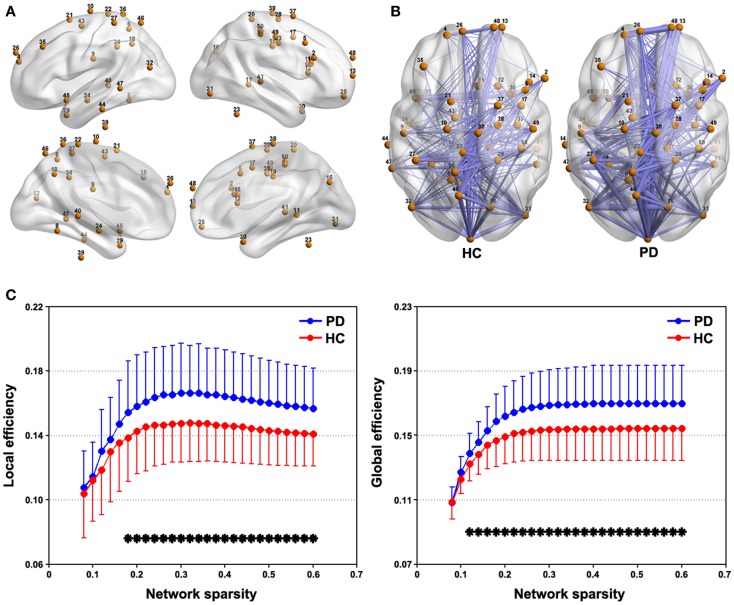
**Between-group differences in network efficiency**. **(A)** Brain surface representation of the 50 ROIs showing abnormal connectivity in PD. **(B)** mean connectivity patterns among the 50 ROIs for the PD and NC groups that were thresholded at a sparsity = 0.3. **(C)** Local and global efficiency the 50-ROI network in the PD and HC groups as a function of sparsity. *P < 0.05, FDR corrected.

Figure 3A represents spatial locations of the 50 sphere ROIs (radius = 6 mm) that exhibited discoordination in PD patients in the brain surface and Figure 3B shows the mean connectivity patterns among these ROIs for each group (sparsity = 0.3). This tremor-related network exhibited typical features of small-world topology for both the PD and HC groups, that is, compared with matched random networks, they had larger local efficiency and almost identical global efficiency (Figure S1 in Supplementary Material). Further between-group comparison revealed significantly (*P* < 0.05, FDR corrected) increased local and global efficiency over a wide range of sparsity threshold in the patients compared with HCs (Figure 3). As for the AUC, the patients also showed higher local (*P* = 0.022) and global (*P* = 0.013) efficiency than the HCs.

### Brain–behavior correlation

**Figure 4:**
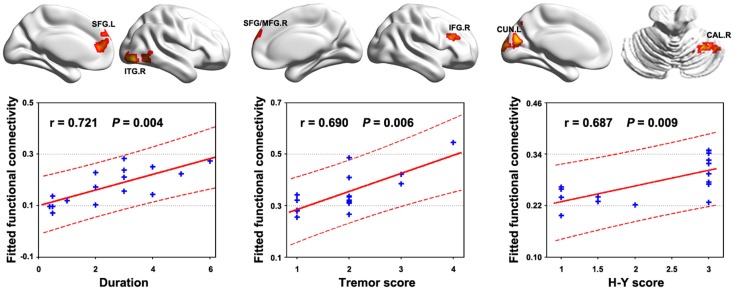
**Relationship between functional connectivity and clinical variables in PD patients**. Significant correlations (*P* < 0.05, FDR corrected) of multiple functional connectivities were observed with the behavior performance of the patients. The figure shows the most significant correlations for the duration, tremor, and H–Y score. All the detected correlations are listed in Table S1 in Supplementary Material.

**Figure 5:**
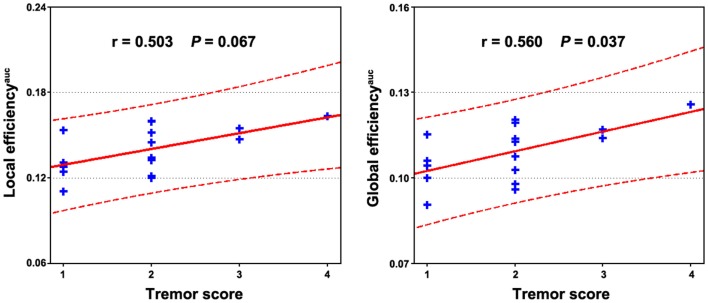
**Relationship between network efficiency and clinical variables in PD patients**. Significantly positive correlations were observed between the AUC of local and global efficiency and tremor score of the patients.

Multiple functional connectivities were observed to be correlated with the patients’ clinical variables (Table S1 in Supplementary Material) after controlling for age, gender, and head motion (*P* < 0.05, FDR corrected). Figure 4 shows the most significant correlations for the duration, tremor, and H–Y score. No correlations were observed for the UPDRS scores. Significant correlations were also observed between local and global efficiency and tremor score for the patients (Figure 5).

### Sensitivity and specificity of abnormal connectivity in differentiating the patients from HCs

**Figure 6:**
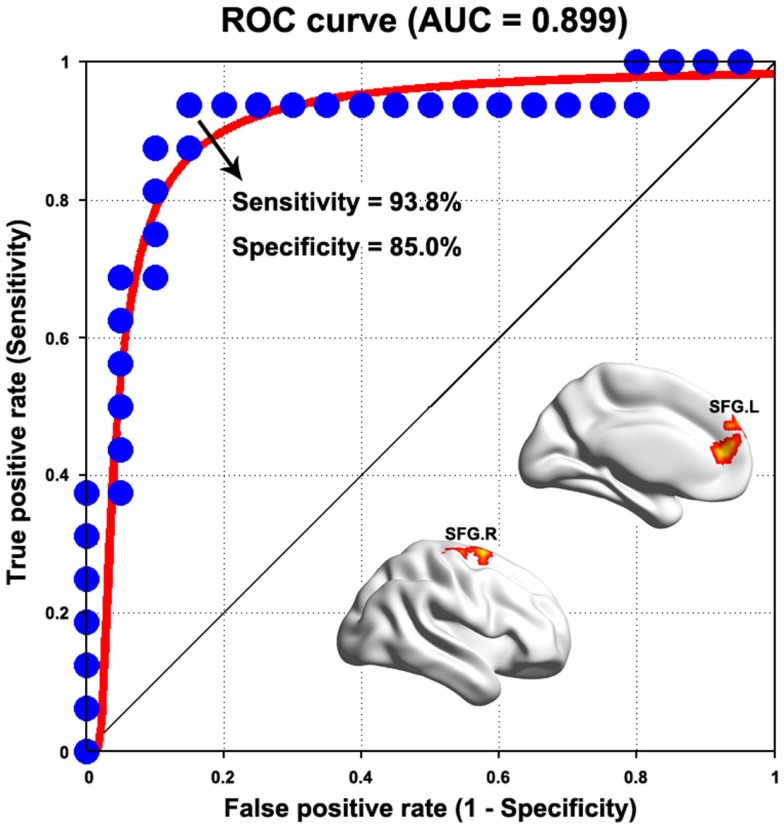
**Receiver operating characteristic curve for distinguishing PD patients from healthy controls as a function of varying functional connectivity between the bilateral superior frontal gyrus (SFG)**. See Table S1 in Supplementary Material for detailed classification information for weighted degree centrality and other functional connectivities.

We found that most of the above-mentioned alterations in the PD group (WDC and functional connectivity) showed fair to good discriminative performances in distinguishing the patients from HCs (Table S1 in Supplementary Material). Figure 6 presents the ROC of functional connectivity between the bilateral superior frontal gyri, which exhibited the highest power (AUC = 0.899, *P* < 10^-12^, 95% CI area = 0.788–1.000), with a maximum sensitivity of 93.8% and a specificity of 85.0%. As such, 15 of the 16 patients with PD and 17 of the 20 HCs were classified correctly.

### Altered GM volume in PD with tremor

Compared with the HCs, the PD patients exhibited significant GM changes in multiple regions (Table S2 in Supplementary Material). Specifically, decreased GM volumes were found in the parietal (right angular gyrus, superior and inferior parietal lobule, and bilateral precuneus), temporal (right superior and middle temporal gyri, parahippocampal gyrus, hippocampus, amygdale, and fusiform gyrus), frontal (right inferior frontal gyrus and rectal gyrus), occipital (left middle occipital gyrus and right lingual gyrus) regions, and the bilateral insula. In contrast, increased GM volume was observed predominantly in the right cerebellum.

**Figure 7:**
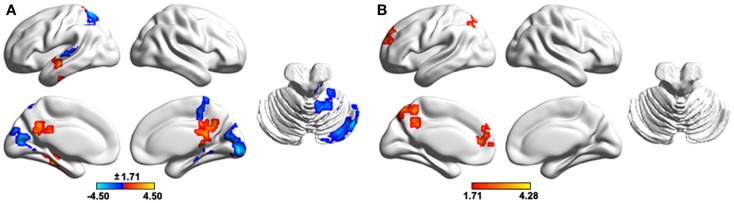
**The effects of regional gray matter volume on the functional results of weighted degree centrality**. **(A)**, Spatial correlation between regional gray matter volume and weighted degree centrality. **(B)**, Between- group differences in weighted degree centrality after controlling for regional gray matter volume. The results were obtained in one general linear model, with weighted degree centrality as the dependent variable, group status (0 or 1) and voxel-specific gray matter volume as independent variables, and gender, age, and head motion as unconcerned covariates. The results were mapped into the brain surface using the BrainNet viewer (http://www.nitrc.org/projects/bnv/).

To test the effects of altered GM volume on between-group differences in functional WDC, individual GM volume maps were treated as extra covariates in a voxel-wise manner during the between-group WDC comparison. The results demonstrated that the structural GM volume showed significant correlations with functional WDC in multiple regions (Figure 7A), largely accounting for the between-group differences in WDC (Figure 7B). Only two clusters, the left SFG and precuneus, survived after controlling the GM volume that increased WDC in the patients.

## Discussion

The current study investigated abnormal functional connectivity networks in PD patients with tremor via a novel combination of voxel-wise centrality, seed-based functional connectivity, and network efficiency analyses. The main results can be summarized as follows: (i) a spatially distributed discoordination network was outlined in PD with tremor that encompassed the cortical (sensorimotor, DMN, prefrontal, and occipital cortices) and subcortical (basal ganglia and thalamus) regions, cerebellum, and brainstem; (ii) the altered connectivity network was highly relevant to the patients’ clinical expressions and exhibited a clinically relevant power in distinguishing the patients from HCs; and (iii) multiple brain regions showed structural changes of GM volume in the patients and the morphological changes largely accounted for the functional connectivity abnormalities.

### Distributed network abnormalities in PD with tremor

Perhaps, the most important advancement of the current work relative to previous studies is that we provided a full delineation of abnormal functional connectivity networks in a specific PD state (i.e., PD with tremor) by searching the entire brain connectome at a refined voxel level. Focusing on focal brain regions or single neural circuit, previous studies documented structural and/or functional changes in PD with tremor (Kassubek et al., 2002; Helmich et al., 2012; Prodoehl et al., 2013). Indeed, increasing evidence has manifested that the human brain is, as a whole, an interconnected complex network. Consistent with this notion, experimental and simulation studies have collectively demonstrated that focal brain lesions not only influence local brain architecture but also spread their effects to other, even distant, brain regions (Alstott et al., 2009; Nomura et al., 2010; Gratton et al., 2012). Inspired by these findings, we speculate that, in addition to these already identified brain sites/circuits, there may exist a spatially more extensive network associated with PD patients with tremor. This was clearly demonstrated in the present study.

The identified abnormal network was involved in several subcortical regions (e.g., basal ganglia and thalamus), the cerebellum, and the brainstem (midbrain and pons). It is not surprising to observe that these sites show altered functional connectivities due to their high relevance in tremor generation in PD, as proposed by previous models (Deuschl et al., 2000; Helmich et al., 2011, 2012; Wu and Hallett, 2013). In addition to these well-established sites, many cortical areas were identified within the network that was predominantly located in sensorimotor cortices (e.g., postcentral gyrus, supplementary motor area, and paracentral lobule) and DMN (e.g., left precuneus, inferior parietal lobule, and middle temporal gyrus). Previous studies have demonstrated that rhythmic oscillations within motor- (e.g., primary motor cortex and cingulate/supplementary motor area) and sensory- (e.g., secondary somatosensory cortex and posterior parietal cortex) related regions are associated with parkinsonian tremor (Volkmann et al., 1996; Timmermann et al., 2003). Indeed, the cerebral motor cortex (e.g., primary motor cortex) has been demonstrated to be a convergence of distinct subcortical–cortical neural circuits (Helmich et al., 2012) that play important roles in tremor. Therefore, it is reasonable to observe abnormal functional connectivity associated with sensorimotor cortices in PD patients with tremor. As for the DMN, previous studies have shown that the DMN regions are structurally interconnected (Greicius et al., 2009; Teipel et al., 2010) and functionally coherent in their brain activity to promote multiple cognitive processes (Greicius et al., 2003; Mason et al., 2007). In PD patients, disturbances of the DMN have been reported to be accompanied by various cognitive deficits, such as recognition memory (Ibarretxe-Bilbao et al., 2011), motor working memory (Rottschy et al., 2013), and motor learning (Argyelan et al., 2008). In our study, we observed that the DMN exhibited abnormal functional connectivity in PD patients. Presumably, these abnormalities may also relate to cognitive deficits, such as impaired working memory in PD (Moustafa et al., 2013). Notably, the patients in the current study were cognitively intact in terms of the MMSE score. This implies that altered network organization might be an early predictive sign of cognitive dysfunction with the progress of the disease, which could be examined in future longitudinal studies. We also detected several prefrontal (e.g., superior, middle, and inferior frontal gyri) and occipital (e.g., calcarine and cuneus) areas showing abnormal functional connectivity in PD. To our knowledge, few studies have examined the roles of these regions in PD, although their deficiencies have been reported in PD (Niethammer et al., 2012). Thus, it is difficult to speculate further on these findings, which should be studied more deeply in the future. Overall, we demonstrated a widely distributed network associated with PD patients with tremor.

### Hyper connectivity in PD with tremor

We identified a disorganized network in which almost all the connections exhibited increased connectivity strength in PD patients with tremor compared with HCs. Previous studies have documented regional hypermetabolism in the basal ganglia, cerebellum, dorsal pons, and primary motor cortex (Mure et al., 2011) and functional connectivity increases within the cerebello-thalamic circuit (Helmich et al., 2011) in tremor PD. These results suggest that overheated functional activities may be common in PD with tremor, consistent with our findings. Moreover, this study expanded previous findings by demonstrating that the hyper connectivity spread over the entire brain in PD with tremor. We further investigated the global properties of these hot-wiring connectivities and found increased network efficiency in the patients relative to HCs.

Generally, the pathophysiology of PD is attributed to the dysfunction of the basal ganglia circuit triggered by deficits in dopaminergic nigrostriatal neurons. Several studies have demonstrated that the dopamine depletion impairs interregional functional connectivity (Nagano-Saito et al., 2008; Wu et al., 2009) and information processing efficiency of resting-state brain networks (Achard and Bullmore, 2007; Carbonell et al., 2014). In patients with PD, decreased interregional connectivity (Luo et al., 2014) and network efficiency do have been observed (Woerner et al., 2009). However, the present study found that there were a large number of connections that exhibited increased functional connectivity in PD patients with tremor compared with HCs and the efficiency among these connections was increased in the patients. The major reason for these discrepancies may be that the current study focused on a unique and characteristic disease state in PD, which was clinically characterized by resting tremor. Although there is also dopaminergic nigrostriatal neurons loss in PD patients with tremor, many clinical studies have shown that only dopamine depletion fails in completely accounting for the symptoms of resting tremor in patients with PD (Helmich et al., 2011). Particularly, clinical studies have found that the level of tremor severity is independent of the amount of dopamine deficiency and is often insensitive to dopamine treatment. Moreover, post-mortem studies also showed that PD patients with tremor have less dopaminergic dysfunction than non-tremor PD patients (Helmich et al., 2012). These findings indicate the existence of other mechanisms that underline tremor PD. Indeed, there are many previous studies that showed elevated GM concentration (Kassubek et al., 2002), regional hypermetabolism (Kassubek et al., 2001) and increased functional connectivity (Helmich et al., 2012) in tremor PD compared with HCs. These findings together with the current results are in correspondence with the notion that there exists a cerebral compensation for pathophysiological alterations in PD patients with tremor (Hallett and Khoshbin, 1980; Rivlin-Etzion et al., 2006). It should be noted that there are few studies that have directly examined the relationship between resting-state networks and neuotransmitters reduction in PD patients with tremor, which will be an important direction for future studies to deepen our understanding of the origin of tremor in PD.

Intriguingly, we found that the increased functional connectivities and network efficiency were positively correlated with the clinical expressions (e.g., tremor level) of the patients. In other words, the more serious the tremor, the higher the strength of these functional connectivities and network efficiency. These correlations indicate the effectiveness of the detected alterations in capturing clinical expressions in PD with tremor and further support the compensatory interpretation. However, it should be pointed out that the compensatory notion in PD with tremor is still speculative currently, thereby, more studies are required on this issue. Finally, the classification analysis showed that the abnormal functional connectivities could distinguish the patients from HCs with clinically relevant discriminative power, indicating the potential of these abnormalities to serve as biomarkers for the disease. Of note, we found that the abnormal functional connectivities exhibiting high relevance with clinical performance and discriminative power were primarily related to cortical, rather than to well-recognized subcortical/cerebellum regions. This finding highlights the important roles of cortical regions in understanding tremor PD and suggests that more attention should be shifted in this direction in future.

In addition to these increased functional connectivities, we also found that there were several decreased functional connectivities in the patients, which may reflect a genuine consequence of the pathological damage of PD to brain function. Neurochemically, PD is generally characterized by an important neurotransmitter depletion, a feature that will lead to a natural assumption that PD is associated with reduced connectivity of numerous connections from a neurochemical perspective. In contrast, we found that there were numerous connections that exhibited increased connectivity strengths in the patients compared with HCs. Although the neural mechanism underlying so many increased connections in PD patients with tremor is unclear currently, as we have discussed above, our findings are consistent with numerous previous studies that showed elevated GM concentration (Kassubek et al., 2002) regional hypermetabolism (Kassubek et al., 2001) and functional connectivity increase (Helmich et al., 2012) in tremor PD. Presumably, these increases may reflect the cerebral compensation for pathophysiological changes of PD (Rivlin-Etzion et al., 2006), which is dominant (versus neurotransmitter depletion) at this specific stage of PD. Of note, there are other possibilities. For example, the current pilot study only included a relative small sample size, which may limit the power to detect more subtle decreases of functional connectivity. Future studies are thus required to provide deeper insights into this issue by recruiting a large cohort of patients. Additionally, the current findings were derived from R-fMRI, which measures spontaneous brain activity. Whether similar findings hold when the patients are engaging in cognitive tasks is an interesting topic in future although the brain’s functional network architecture during task performance is strongly shaped by an intrinsic network architecture during rest (Cole et al., 2014).

### GM atrophy in PD with tremor

Beyond functional abnormalities, we also studied structural GM volume changes in these PD patients. There are many studies investigating GM volume in PD patients (Brenneis et al., 2007; Jubault et al., 2011; Pereira et al., 2012); however, the findings are far from a consensus (Lin et al., 2013). One possible factor accounting for such inconsistency is the mixture of different PD symptom dimensions that demonstrate distinct patterns of GM volume alterations (Benninger et al., 2009). Focusing on single-domain PD patients, Kassubek et al. studied a specific region of interest, the posterior ventral lateral thalamus in PD patients with tremor, and found increased GM volume compared with HCs (Kassubek et al., 2002). Using an unbiased VBM method, here we detailed GM alterations over the entire cortical mantle in PD with tremor. We found that PD patients with tremor were associated with widespread GM volume reductions in multiple regions of cortical (e.g., right inferior frontal gyrus, bilateral middle temporal gyrus, and left middle occipital gyrus) and limbic/subcortical (e.g., right hippocampus, parahippocampus, amygdale, and bilateral insula) sites. The volumetric reductions might reflect neural degeneration owing to the pathological damage of the disease (Benninger et al., 2009). More importantly, we found that the volumetric reductions had significant correlations with the patients’ clinical expressions, suggesting a neuroanatomical significance of these abnormalities in monitoring the progression of the disease. In addition, we also found that the cerebellum exhibited increased GM volume in the PD patients. Generally, GM volume increase may be due to neuronal hypertrophy or higher neuron density (Kassubek et al., 2002), which reflect brain neuroplasticity (Brosh and Barkai, 2004) to respond external environmental cues, experience, behavior, injury, or disease (Ludlow et al., 2008). Thus, we speculate that the increased cerebellar GM volume observed in the PD patients with tremor may relate to the neuroplasticity due to long-term pathological and/or behavior changes in the patients. Of note, we did not detect GM changes in the thalamus in this dataset, which may be due to the small sample size.

Previous studies have shown that regional GM loss significantly contributes to functional abnormalities in patients with Alzheimer’s disease (He et al., 2007). Analogously, we also observed that functional abnormalities can be largely accounted for by GM volume changes in PD patients with tremor. This implies a structural basis of the observed functional abnormalities in this specific PD state and suggests that more attention should be given to the impacts of regional morphological changes on functional results in future studies of neurodegenerative diseases (He et al., 2007). Notably, the functional abnormalities of two clusters (i.e., left SFG and precuneus) were independent of GM volume alterations, suggesting their important roles in understanding how the disease exerts influence on brain function.

### Limitations

Several issues need to be addressed in future research. First, the small sample size limited the conclusions that we can draw; thus, a large number of participants should be included in future studies. Second, in this study, only *post hoc* motion correction methods were used to correct for motion artifacts by combining individual- and group-level strategies. However, despite of our efforts to attenuate head-motion effects as much as possible, we cannot exclude the possibility of residual head-motion effects on our results. Further studies are required to reproduce our findings using new methods with the development of this field. Third, although this study identified the abnormally organized network related to PD with tremor, it did not answer how this abnormal component reshapes whole-brain network topology, such as large-scale small-world organization and intermediate-scale community structure (Gottlich et al., 2013; Olde Dubbelink et al., 2014). Fourth, the present study focused on functional brain networks in PD with tremor. Recent studies document tight relationships in the connectivity patterns between structural and functional brain networks (Honey et al., 2009; van den Heuvel et al., 2009; Hermundstad et al., 2013) and the structure–function coupling was found to be disrupted under the pathological condition (Zhang et al., 2011b). Therefore, combining multimodal neuroimaging datasets (e.g., task fMRI, positron emission tomography, arterial spin labeling, and diffusion tensor imaging) will provide more informative insight into neural response to task demands, neurotransmitters disruption, physiological basis, structure basis, and the influences of the disease on the collective behavior of the brain. Fifth, the present study outlined whole-brain functional network abnormalities in PD patients with tremor. In the future, it will be important to determine the similarities and differences in the abnormal network patterns among PD patients dominated by different clinical symptoms. Sixth, the current dataset was obtained on a 1.5 T MRI scanner, which is less sensitive in the detection of resting-state brain networks. The findings observed here should be further validated using high field magnet scanner. Finally, the patients recruited in the current study were under an off-medicine condition. Whether the observed changes are normalized during the drug effect should be further considered.

## Conclusion

Combining network centrality, seed-based functional connectivity, and network efficiency analyses, the present study provides a full map of abnormal connectivity networks in PD with tremor that is distributed over cortical, subcortical, cerebellum, and brainstem sites. These altered functional connectivities correlated with the clinical performance of the patients and exhibited clinically relevant discriminative power in distinguishing the patients from HCs. Moreover, the functional changes were explained to a great extent by abnormal GM volume, indicating a structural basis of functional alterations. These findings have important implications in understanding the neural substrates underlying the specific type of PD with tremor patient.

## Conflict of Interest Statement

The authors declare that the research was conducted in the absence of any commercial or financial relationships that could be construed as a potential conflict of interest.

## Supplementary Material

The Supplementary Material for this article can be found online at http://www.frontiersin.org/Journal/10.3389/fnagi.2015.00006/abstract

## Footnotes

^1^http://www.nitrc.org/projects/gretna/

^2^http://www.fil.ion.ucl.ac.uk/spm/software/spm8/

^3^http://dbm.neuro.uni-jena.de/software/

^4^http://www.mathworks.com/matlabcentral/fileexchange/19950

## References

[B1] Achard S. Bullmore E. (2007). Efficiency and cost of economical brain functional networks. PLoS Comput. Biol. 3:e17. 10.1371/journal.pcbi.0030017 17274684 PMC1794324

[B2] Alstott J. Breakspear M. Hagmann P. Cammoun L. Sporns O. (2009). Modeling the impact of lesions in the human brain. PLoS Comput. Biol. 5:e1000408. 10.1371/journal.pcbi.1000408 19521503 PMC2688028

[B3] Argyelan M. Carbon M. Ghilardi M. F. Feigin A. Mattis P. Tang C. (2008). Dopaminergic suppression of brain deactivation responses during sequence learning. J. Neurosci. 28, 10687–10695. 10.1523/JNEUROSCI.2933-08.2008 18923044 PMC4617653

[B4] Benninger D. H. Thees S. Kollias S. S. Bassetti C. L. Waldvogel D. (2009). Morphological differences in Parkinson’s disease with and without rest tremor. J. Neurol. 256, 256–263. 10.1007/s00415-009-0092-2 19219572

[B5] Biswal B. Yetkin F. Z. Haughton V. M. Hyde J. S. (1995). Functional connectivity in the motor cortex of resting human brain using echo-planar MRI. Magn. Reson. Med. 34, 537–541. 10.1002/mrm.1910340409 8524021

[B6] Brenneis C. Egger K. Scherfler C. Seppi K. Schocke M. Poewe W. (2007). Progression of brain atrophy in multiple system atrophy. J. Neurol. 254, 191–196. 10.1007/s00415-006-0325-6 17334661

[B7] Brosh I. Barkai E. (2004). Learning-induced long-term synaptic modifications in the olfactory cortex. Curr. Neurovasc. Res. 1, 389–395. 10.2174/1567202043362090 16181087

[B8] Buckner R. L. Sepulcre J. Talukdar T. Krienen F. M. Liu H. Hedden T. (2009). Cortical hubs revealed by intrinsic functional connectivity: mapping, assessment of stability, and relation to Alzheimer’s disease. J. Neurosci. 29, 1860–1873. 10.1523/JNEUROSCI.5062-08.2009 19211893 PMC2750039

[B9] Bullmore E. Sporns O. (2009). Complex brain networks: graph theoretical analysis of structural and functional systems. Nat. Rev. Neurosci. 10, 186–198 10.1038/nrn2575 19190637

[B10] Cao H. Plichta M. M. Schafer A. Haddad L. Grimm O. Schneider M. (2014). Test-retest reliability of fMRI-based graph theoretical properties during working memory, emotion processing, and resting state. Neuroimage 84, 888–900. 10.1016/j.neuroimage.2013.09.013 24055506

[B11] Carbonell F. Nagano-Saito A. Leyton M. Cisek P. Benkelfat C. He Y. (2014). Dopamine precursor depletion impairs structure and efficiency of resting state brain functional networks. Neuropharmacology 84, 90–100. 10.1016/j.neuropharm.2013.12.021 24412649

[B12] Cole M. W. Bassett D. S. Power J. D. Braver T. S. Petersen S. E. (2014). Intrinsic and task-evoked network architectures of the human brain. Neuron 83, 238–251. 10.1016/j.neuron.2014.05.014 24991964 PMC4082806

[B13] Deuschl G. Raethjen J. Baron R. Lindemann M. Wilms H. Krack P. (2000). The pathophysiology of parkinsonian tremor: a review. J. Neurol. 247(Suppl. 5), V33–V48. 10.1007/PL00007781 11081802

[B14] Di Martino A. Zuo X. N. Kelly C. Grzadzinski R. Mennes M. Schvarcz A. (2013). Shared and distinct intrinsic functional network centrality in autism and attention-deficit/hyperactivity disorder. Biol. Psychiatry 74, 623–632. 10.1016/j.biopsych.2013.02.011 23541632 PMC4508007

[B15] Fair D. A. Nigg J. T. Iyer S. Bathula D. Mills K. L. Dosenbach N. U. (2012). Distinct neural signatures detected for ADHD subtypes after controlling for micro-movements in resting state functional connectivity MRI data. Front. Syst. Neurosci. 6:80. 10.3389/fnsys.2012.00080 23382713 PMC3563110

[B16] Fishman P. S. (2008). Paradoxical aspects of parkinsonian tremor. Mov. Disord. 23, 168–173 10.1002/mds.21736 17973325

[B17] Fox M. D. Zhang D. Snyder A. Z. Raichle M. E. (2009). The global signal and observed anticorrelated resting state brain networks. J. Neurophysiol. 101, 3270–3283. 10.1152/jn.90777.2008 19339462 PMC2694109

[B18] Friston K. J. Williams S. Howard R. Frackowiak R. S. Turner R. (1996). Movement-related effects in fMRI time-series. Magn. Reson. Med. 35, 346–355. 10.1002/mrm.1910350312 8699946

[B19] Gibb W. R. Lees A. J. (1988). The relevance of the Lewy body to the pathogenesis of idiopathic Parkinson’s disease. J. Neurol. Neurosurg. Psychiatr. 51, 745–752. 10.1136/jnnp.51.6.745 2841426 PMC1033142

[B20] Gottlich M. Munte T. F. Heldmann M. Kasten M. Hagenah J. Kramer U. M. (2013). Altered resting state brain networks in Parkinson’s disease. PLoS ONE 8:e77336. 10.1371/journal.pone.0077336 24204812 PMC3810472

[B21] Gratton C. Nomura E. M. Perez F. D’Esposito M. (2012). Focal brain lesions to critical locations cause widespread disruption of the modular organization of the brain. J. Cogn. Neurosci. 24, 1275–1285. 10.1162/jocn_a_00222 22401285 PMC3575518

[B22] Greicius M. D. Krasnow B. Reiss A. L. Menon V. (2003). Functional connectivity in the resting brain: a network analysis of the default mode hypothesis. Proc. Natl. Acad. Sci. U. S. A. 100, 253–258. 10.1073/pnas.0135058100 12506194 PMC140943

[B23] Greicius M. D. Supekar K. Menon V. Dougherty R. F. (2009). Resting-state functional connectivity reflects structural connectivity in the default mode network. Cereb. Cortex 19, 72–78. 10.1093/cercor/bhn059 18403396 PMC2605172

[B24] Hacker C. D. Perlmutter J. S. Criswell S. R. Ances B. M. Snyder A. Z. (2012). Resting state functional connectivity of the striatum in Parkinson’s disease. Brain 135(Pt 12), 3699–3711. 10.1093/brain/aws281 23195207 PMC3525055

[B25] Hallett M. Khoshbin S. (1980). A physiological mechanism of bradykinesia. Brain 103, 301–314 10.1093/brain/103.2.301 7397480

[B26] He Y. Dagher A. Chen Z. Charil A. Zijdenbos A. Worsley K. (2009). Impaired small-world efficiency in structural cortical networks in multiple sclerosis associated with white matter lesion load. Brain 132, 3366–3379. 10.1093/brain/awp089 19439423 PMC2792366

[B27] He Y. Evans A. (2010). Graph theoretical modeling of brain connectivity. Curr. Opin. Neurol. 23, 341–350. 10.1097/WCO.0b013e32833aa567 20581686

[B28] He Y. Wang L. Zang Y. F. Tian L. X. Zhang X. Q. Li K. C. (2007). Regional coherence changes in the early stages of Alzheimer’s disease: a combined structural and resting-state functional MRI study. Neuroimage 35, 488–500. 10.1016/j.neuroimage.2006.11.042 17254803

[B29] Helmich R. C. Hallett M. Deuschl G. Toni I. Bloem B. R. (2012). Cerebral causes and consequences of parkinsonian resting tremor: a tale of two circuits? Brain 135(Pt 11), 3206–3226. 10.1093/brain/aws023 22382359 PMC3501966

[B30] Helmich R. C. Janssen M. J. Oyen W. J. Bloem B. R. Toni I. (2011). Pallidal dysfunction drives a cerebellothalamic circuit into Parkinson tremor. Ann. Neurol. 69, 269–281. 10.1002/ana.22361 21387372

[B31] Hermundstad A. M. Bassett D. S. Brown K. S. Aminoff E. M. Clewett D. Freeman S. (2013). Structural foundations of resting-state and task-based functional connectivity in the human brain. Proc. Natl. Acad. Sci. U. S. A. 110, 6169–6174. 10.1073/pnas.1219562110 23530246 PMC3625268

[B32] Honey C. J. Sporns O. Cammoun L. Gigandet X. Thiran J. P. Meuli R. (2009). Predicting human resting-state functional connectivity from structural connectivity. Proc. Natl. Acad. Sci. U. S. A. 106, 2035–2040. 10.1073/pnas.0811168106 19188601 PMC2634800

[B33] Ibarretxe-Bilbao N. Zarei M. Junque C. Marti M. J. Segura B. Vendrell P. (2011). Dysfunctions of cerebral networks precede recognition memory deficits in early Parkinson’s disease. Neuroimage 57, 589–597. 10.1016/j.neuroimage.2011.04.049 21554963

[B34] Jankovic J. McDermott M. Carter J. Gauthier S. Goetz C. Golbe L. (1990). Variable expression of Parkinson’s disease: a base-line analysis of the DATATOP cohort. The Parkinson Study Group. Neurology 40, 1529–1534. 10.1212/WNL.40.10.1529 2215943

[B35] Jellinger K. A. (1999). Post mortem studies in Parkinson’s disease – is it possible to detect brain areas for specific symptoms? J. Neural. Transm. Suppl. 56, 1–29. 10.1007/978-3-7091-6360-3_1 10370901

[B36] Jubault T. Gagnon J. F. Karama S. Ptito A. Lafontaine A. L. Evans A. C. (2011). Patterns of cortical thickness and surface area in early Parkinson’s disease. Neuroimage 55, 462–467. 10.1016/j.neuroimage.2010.12.043 21184830

[B37] Kassubek J. Juengling F. D. Hellwig B. Knauff M. Spreer J. Lucking C. H. (2001). Hypermetabolism in the ventrolateral thalamus in unilateral Parkinsonian resting tremor: a positron emission tomography study. Neurosci. Lett. 304, 17–20. 10.1016/S0304-3940(01)01737-2 11335044

[B38] Kassubek J. Juengling F. D. Hellwig B. Spreer J. Lucking C. H. (2002). Thalamic gray matter changes in unilateral Parkinsonian resting tremor: a voxel-based morphometric analysis of 3-dimensional magnetic resonance imaging. Neurosci. Lett. 323, 29–32. 10.1016/S0304-3940(02)00111-8 11911983

[B39] Latora V. Marchiori M. (2003). Economic small-world behavior in weighted networks. Eur. Phys. J. B 32, 249–263 10.1140/epjb/e2003-00095-5

[B40] Ledberg A. Akerman S. Roland P. E. (1998). Estimation of the probabilities of 3D clusters in functional brain images. Neuroimage 8, 113–128 10.1006/nimg.1998.0336 9740755

[B41] Lees A. J. (2007). Unresolved issues relating to the shaking palsy on the celebration of James Parkinson’s 250th birthday. Mov. Disord. 22(Suppl. 17), S327–S334. 10.1002/mds.21684 18175393

[B42] Lees A. J. Hardy J. Revesz T. (2009). Parkinson’s disease. Lancet 373, 2055–2066. 10.1016/S0140-6736(09)60492-X 19524782

[B43] Lin C. H. Chen C. M. Lu M. K. Tsai C. H. Chiou J. C. Liao J. R. (2013). VBM reveals brain volume differences between Parkinson’s disease and essential tremor patients. Front. Hum. Neurosci. 7:247. 10.3389/fnhum.2013.00247 23785322 PMC3682128

[B44] Ludlow C. L. Hoit J. Kent R. Ramig L. O. Shrivastav R. Strand E. (2008). Translating principles of neural plasticity into research on speech motor control recovery and rehabilitation. J. Speech Lang. Hear. Res. 51, S240–S258. 10.1044/1092-4388(2008/019) 18230849 PMC2364711

[B45] Luo C. Song W. Chen Q. Zheng Z. Chen K. Cao B. (2014). Reduced functional connectivity in early-stage drug-naive Parkinson’s disease: a resting-state fMRI study. Neurobiol. Aging 35, 431–441. 10.1016/j.neurobiolaging.2013.08.018 24074808

[B46] Maslov S. Sneppen K. (2002). Specificity and stability in topology of protein networks. Science 296, 910–913. 10.1126/science.1065103 11988575

[B47] Mason M. F. Norton M. I. Van Horn J. D. Wegner D. M. Grafton S. T. Macrae C. N. (2007). Wandering minds: the default network and stimulus-independent thought. Science 315, 393–395 10.1126/science.1131295 17234951 PMC1821121

[B48] Milo R. Shen-Orr S. Itzkovitz S. Kashtan N. Chklovskii D. Alon U. (2002). Network motifs: simple building blocks of complex networks. Science 298, 824–827 10.1126/science.298.5594.824 12399590

[B49] Moustafa A. A. Bell P. Eissa A. M. Hewedi D. H. (2013). The effects of clinical motor variables and medication dosage on working memory in Parkinson’s disease. Brain Cogn. 82, 137–145. 10.1016/j.bandc.2013.04.001 23660434

[B50] Mure H. Hirano S. Tang C. C. Isaias I. U. Antonini A. Ma Y. (2011). Parkinson’s disease tremor-related metabolic network: characterization, progression, and treatment effects. Neuroimage 54, 1244–1253. 10.1016/j.neuroimage.2010.09.028 20851193 PMC2997135

[B51] Murphy K. Birn R. M. Handwerker D. A. Jones T. B. Bandettini P. A. (2009). The impact of global signal regression on resting state correlations: are anti-correlated networks introduced? Neuroimage 44, 893–905. 10.1016/j.neuroimage.2008.09.036 18976716 PMC2750906

[B52] Nagano-Saito A. Leyton M. Monchi O. Goldberg Y. K. He Y. Dagher A. (2008). Dopamine depletion impairs frontostriatal functional connectivity during a set-shifting task. J. Neurosci. 28, 3697–3706. 10.1523/JNEUROSCI.3921-07.2008 18385328 PMC6671089

[B53] Niethammer M. Feigin A. Eidelberg D. (2012). Functional neuroimaging in Parkinson’s disease. Cold Spring Harb. Perspect. Med. 2, a009274. 10.1101/cshperspect.a009274 22553499 PMC3331691

[B54] Nomura E. M. Gratton C. Visser R. M. Kayser A. Perez F. D’Esposito M. (2010). Double dissociation of two cognitive control networks in patients with focal brain lesions. Proc. Natl. Acad. Sci. U. S. A. 107, 12017–12022. 10.1073/pnas.1002431107 20547857 PMC2900657

[B55] Olde Dubbelink K. T. Hillebrand A. Stoffers D. Deijen J. B. Twisk J. W. Stam C. J. (2014). Disrupted brain network topology in Parkinson’s disease: a longitudinal magnetoencephalography study. Brain 137(Pt 1), 197–207. 10.1093/brain/awt316 24271324

[B56] Paulus W. Jellinger K. (1991). The neuropathologic basis of different clinical subgroups of Parkinson’s disease. J. Neuropathol. Exp. Neurol. 50, 743–755. 10.1097/00005072-199111000-00006 1748881

[B57] Pereira J. B. Ibarretxe-Bilbao N. Marti M. J. Compta Y. Junque C. Bargallo N. (2012). Assessment of cortical degeneration in patients with Parkinson’s disease by voxel-based morphometry, cortical folding, and cortical thickness. Hum. Brain Mapp. 33, 2521–2534. 10.1002/hbm.21378 21898679 PMC6870035

[B58] Prodoehl J. Planetta P. J. Kurani A. S. Comella C. L. Corcos D. M. Vaillancourt D. E. (2013). Differences in brain activation between tremor- and nontremor-dominant Parkinson disease. JAMA Neurol. 70, 100–106. 10.1001/jamaneurol.2013.582 23318516 PMC3645004

[B59] Rivlin-Etzion M. Marmor O. Heimer G. Raz A. Nini A. Bergman H. (2006). Basal ganglia oscillations and pathophysiology of movement disorders. Curr. Opin. Neurobiol. 16, 629–637. 10.1016/j.conb.2006.10.002 17084615

[B60] Rodriguez-Oroz M. C. Jahanshahi M. Krack P. Litvan I. Macias R. Bezard E. (2009). Initial clinical manifestations of Parkinson’s disease: features and pathophysiological mechanisms. Lancet Neurol. 8, 1128–1139. 10.1016/S1474-4422(09)70293-5 19909911

[B61] Rottschy C. Kleiman A. Dogan I. Langner R. Mirzazade S. Kronenbuerger M. (2013). Diminished activation of motor working-memory networks in Parkinson’s disease. PLoS ONE 8:e61786. 10.1371/journal.pone.0061786 23620791 PMC3631252

[B62] Teipel S. J. Bokde A. L. Meindl T. Amaro E. Jr. Soldner J. Reiser M. F. (2010). White matter microstructure underlying default mode network connectivity in the human brain. Neuroimage 49, 2021–2032. 10.1016/j.neuroimage.2009.10.067 19878723

[B63] Timmermann L. Gross J. Dirks M. Volkmann J. Freund H. J. Schnitzler A. (2003). The cerebral oscillatory network of parkinsonian resting tremor. Brain 126(Pt 1), 199–212. 10.1093/brain/awg022 12477707

[B64] Tomasi D. Volkow N. D. (2010). Functional connectivity density mapping. Proc. Natl. Acad. Sci. U. S. A. 107, 9885–9890. 10.1073/pnas.1001414107 20457896 PMC2906909

[B65] Toth C. Rajput M. Rajput A. H. (2004). Anomalies of asymmetry of clinical signs in parkinsonism. Mov. Disord. 19, 151–157. 10.1002/mds.10685 14978669

[B66] van den Heuvel M. P. Mandl R. C. Kahn R. S. Hulshoff Pol H. E. (2009). Functionally linked resting-state networks reflect the underlying structural connectivity architecture of the human brain. Hum. Brain Mapp. 30, 3127–3141. 10.1002/hbm.20737 19235882 PMC6870902

[B67] Van Dijk K. R. A. Hedden T. Venkataraman A. Evans K. C. Lazar S. W. Buckner R. L. (2010). Intrinsic functional connectivity as a tool for human connectomics: theory, properties, and optimization. J. Neurophysiol. 103, 297–321. 10.1152/jn.00783.2009 19889849 PMC2807224

[B68] Volkmann J. Joliot M. Mogilner A. Ioannides A. A. Lado F. Fazzini E. (1996). Central motor loop oscillations in parkinsonian resting tremor revealed by magnetoencephalography. Neurology 46, 1359–1370. 10.1212/WNL.46.5.1359 8628483

[B69] Wang J. H. Zuo X. N. Gohel S. Milham M. P. Biswal B. B. He Y. (2011). Graph theoretical analysis of functional brain networks: test-retest evaluation on short- and long-term resting-state functional MRI data. PLoS ONE 6:e21976. 10.1371/journal.pone.0021976 21818285 PMC3139595

[B70] Weissenbacher A. Kasess C. Gerstl F. Lanzenberger R. Moser E. Windischberger C. (2009). Correlations and anticorrelations in resting-state functional connectivity MRI: a quantitative comparison of preprocessing strategies. Neuroimage 47, 1408–1416. 10.1016/j.neuroimage.2009.05.005 19442749

[B71] Woerner L. Espinosa J. Bourne S. O’Toole M. Ingersoll G. L. (2009). Project (inverted exclamation mark)EXITO!: success through diversity and universality for outcomes improvement among Hispanic home care patients. Nurs. Outlook 57, 266–273. 10.1016/j.outlook.2009.02.001 19789004

[B72] Wu T. Hallett M. (2013). The cerebellum in Parkinson’s disease. Brain 136(Pt 3), 696–709 10.1093/brain/aws360 23404337 PMC7273201

[B73] Wu T. Wang L. Chen Y. Zhao C. Li K. Chan P. (2009). Changes of functional connectivity of the motor network in the resting state in Parkinson’s disease. Neurosci. Lett. 460, 6–10. 10.1016/j.neulet.2009.05.046 19463891

[B74] Yan C. G. Cheung B. Kelly C. Colcombe S. Craddock R. C. Di Martino A. (2013). A comprehensive assessment of regional variation in the impact of head micromovements on functional connectomics. Neuroimage 76, 183–201. 10.1016/j.neuroimage.2013.03.004 23499792 PMC3896129

[B75] Zhang J. Wang J. Wu Q. Kuang W. Huang X. He Y. (2011a). Disrupted brain connectivity networks in drug-naive, first-episode major depressive disorder. Biol. Psychiatry 70, 334–342. 10.1016/j.biopsych.2011.05.018 21791259

[B76] Zhang Z. Liao W. Chen H. Mantini D. Ding J. R. Xu Q. (2011b). Altered functional-structural coupling of large-scale brain networks in idiopathic generalized epilepsy. Brain 134(Pt 10), 2912–2928. 10.1093/brain/awr223 21975588

[B77] Zuo X. N. Ehmke R. Mennes M. Imperati D. Castellanos F. X. Sporns O. (2012). Network centrality in the human functional connectome. Cereb. Cortex 22, 1862–1875 10.1093/cercor/bhr269 21968567

