# Supplementary material for: Widespread Increase of Functional Connectivity in Parkinson’s Disease with Tremor: A Resting-State fMRI Study
Source: Front Aging Neurosci. 2015 Feb 3;7:6. doi: 10.3389/fnagi.2015.00006 (PMC4315047; doi:10.3389/fnagi.2015.00006)
Supplement: Supplementary file 1 [file Table1.DOC]

**Table S1**. Summary of between-group differences in weighted degree centrality and seed-based functional connectivity and their correlation with clinical performances and classification.

|  | **Between-group difference** | | | **Correlation** | | | **Classification** | |
| --- | --- | --- | --- | --- | --- | --- | --- | --- |
| Regions | Volume (mm^3^) | Peak MNI  (x, y, z) | Direction | Duration | Tremor | H-Y | AUC | *P* |
| **Weighted degree centrality** | | | | | | | | |
| ^1^CereAL.L | 8019 | 0 -51 -3 | PD<HC | - | - | - | 0.602 | 0.146 |
| ^2^MFG/IFG.R | 5481 | 57 24 27 | PD>HC | - | 0.622 | - | 0.687 | 0.020 |
| ^3^CUN/PCUN/CAL.L | 8505 | 0 -99 6 | PD>HC | - | - | - | 0.607 | 0.133 |
| ^4^SFG.L | 7938 | -18 57 24 | PD>HC | - | 0.582 | - | 0.666 | 0.037 |
| ^5^MFG/SFG.R | 10827 | 42 15 42 | PD>HC | - | - | - | 0.743 | 0.002 |
| ^6^PCUN/PoCG.L | 18063 | -15 -54 57 | PD>HC | - | - | - | 0.812 | <10^-3^ |
| ^7^PreCG/PoCG.R | 5022 | 15 -30 51 | PD>HC | - | - | - | 0.668 | 0.035 |
| **Functional connectivity** (seed 1, the left Vermis, center = [0 -51 -3], r = 6 mm, sphere) | | | | | | | | |
| ^8^CereAL.L | 1593 | -24 -54 -15 | PD>HC | - | - | - | 0.748 | 0.002 |
| ^9^PoCG.L | 1026 | -51 -18 27 | PD<HC | - | - | - | 0.865 | <10^-3^ |
| ^10^PreCG.L | 1026 | -18 -15 75 | PD>HC | - | - | - | 0.8301 | <10^-3^ |
| **Functional connectivity** (seed 2, the right IFGtriang, center = [57 24 27], r = 6 mm, sphere) | | | | | | | | |
| ^11^STG.R | 2430 | 51 -42 0 | PD>HC | - | - | - | 0.793 | <10^-3^ |
| ^12^CAU.R | 1971 | 21 18 12 | PD>HC | - | - | - | 0.860 | <10^-3^ |
| ^13^SFG.R | 1485 | 24 63 9 | PD>HC | - | - | - | 0.852 | <10^-3^ |
| ^14^IFGtriang.R | 2349 | 45 21 21 | PD>HC | - | - | - | 0.675 | 0.029 |
| ^15^ACG.R | 1215 | 6 18 21 | PD>HC | - | - | 0.596 | 0.813 | <10^-3^ |
| ^16^CUN.R | 1134 | 15 -75 33 | PD>HC | - | - | - | 0.687 | 0.020 |
| ^17^MFG.R | 3780 | 39 3 48 | PD>HC | - | - | - | 0.816 | <10^-3^ |
| ^18^PCUN.L | 1593 | -9 -57 42 | PD>HC | - | - | - | 0.807 | <10^-3^ |
| ^19^MCG.R | 2106 | 12 -18 39 | PD>HC | - | - | - | 0.779 | <10^-3^ |
| ^20^PoCG.R | 2403 | 21 -39 66 | PD>HC | - | 0.609 | - | 0.829 | <10^-3^ |
| ^21^SFG.L | 1539 | -15 6 66 | PD>HC | - | - | - | 0.779 | <10^-3^ |
| ^22^PCL.L | 2808 | -6 -33 72 | PD>HC | - | - | - | 0.802 | <10^-3^ |
| **Functional connectivity** (seed 3, the left CAL, center = [0 -99 6], r = 6 mm, sphere) | | | | | | | | |
| ^23^CereAL.R | 1971 | 15 -54 -30 | PD>HC | - | - | 0.683 | 0.819 | <10^-3^ |
| ^24^MTG.L | 2376 | -48 -12 -15 | PD>HC | - | - | - | 0.816 | <10^-3^ |
| ^25^SFG.R | 2052 | 21 54 -12 | PD>HC | - | - | - | 0.863 | <10^-3^ |
| ^26^SFGmed.L | 1782 | -6 60 33 | PD>HC | - | - | - | 0.790 | <10^-3^ |
| ^27^PoCG.L | 1296 | -42 -39 63 | PD>HC | - | - | - | 0.759 | 0.001 |
| ^28^SFG.R | 1080 | 21 -12 66 | PD>HC | - | - | - | 0.706 | 0.010 |
| PoCG.R | 1134 | 27 -36 66 | PD>HC | - | - | - | 0.695 | 0.015 |
| **Functional connectivity** (seed 4, the left SFG, center = [-18 57 24], r = 6 mm, sphere) | | | | | | | | |
| ^29^STG.L | 1458 | -30 9,-30 | PD>HC | - | - | - | 0.826 | <10^-3^ |
| ^30^STG.R | 1107 | 30 12 -27 | PD>HC | - | - | 0.565 | 0.778 | <10^-3^ |
| ^31^ITG.R | 3699 | 42 -81 -9 | PD>HC | 0.721 | - | - | 0.778 | <10^-3^ |
| ^32^MTG.L | 1053 | -45 -75 18 | PD>HC | - | - | - | 0.781 | <10^-3^ |
| ^33^PreCG.R | 2295 | 36 -12 42 | PD>HC | - | - | - | 0.786 | <10^-3^ |
| ^34^IPL.L | 2025 | -30 -42 39 | PD>HC | - | - | - | 0.781 | <10^-3^ |
| ^35^MFG.L | 1188 | -36 33 39 | PD>HC | - | - | - | 0.805 | <10^-3^ |
| ^36^PCUN.L | 2646 | -12 -48 72 | PD>HC | - | - | - | 0.761 | 0.001 |
| ^37^SFG.R | 2160 | 21 3 69 | PD>HC | - | - | - | 0.899 | <10^-3^ |
| ^38^MFG.R | 1080 | 6 -18 72 | PD>HC | - | - | - | 0.776 | <10^-3^ |
| PoCG.R | 1674 | 24 -36 72 | PD>HC | - | - | - | 0.771 | 0.001 |
| SFG.L | 1647 | -21 54 24 | PD>HC | - | - | 0.625 | 0.729 | 0.004 |
| **Functional connectivity** (seed 5, the right MFG, center = [42 15 42], r = 6 mm, sphere) | | | | | | | | |
| ^39^Pons | 1080 | -12 -30 -42 | PD<HC | - | - | - | 0.855 | <10^-3^ |
| ^40^Midbrain.L | 1107 | -6 -33 0 | PD<HC | - | - | - | 0.781 | <10^-3^ |
| ^41^STG.R | 1296 | 51 -30 3 | PD>HC | - | - | - | 0.725 | 0.005 |
| ^42^PCUN.L | 1890 | 0 -45 45 | PD>HC | - | - | - | 0.882 | <10^-3^ |
| ^43^SMA.L | 2511 | -12 -6 60 | PD>HC | - | - | - | 0.823 | <10^-3^ |
| IFGtriang.R | 1485 | 51 24 27 | PD>HC | - | 0.690 | - | 0.738 | 0.003 |
| SFG.R | 1053 | 24 63 18 | PD>HC | - | - | - | 0.796 | <10^-3^ |
| **Functional connectivity** (seed 6, the left PCUN, center = [-15 -54 57], r = 6 mm, sphere) | | | | | | | | |
| ^44^ITG.L | 1296 | -63 -27 -24 | PD>HC | - | - | - | 0.837 | <10^-3^ |
| ^45^INS.L | 2079 | -42 9 -15 | PD<HC | - | - | - | 0.803 | <10^-3^ |
| ^46^PCUN.L | 4563 | -9 -66 63 | PD>HC | - | - | - | 0.766 | 0.001 |
| **Functional connectivity** (seed 7, center = [15 -30 51], r = 6 mm, sphere) | | | | | | | | |
| ^47^MTG.L | 1134 | -60 -45 -3 | PD>HC | - | - | - | 0.870 | <10^-3^ |
| ^48^SFG.R | 1080 | 18 63 27 | PD>HC | - | - | - | 0.862 | <10^-3^ |
| ^49^PreCG.R | 2214 | 48 -15 48 | PD>HC | - | - | - | 0.844 | <10^-3^ |
| ^50^PoCG.R | 1836 | 39 -30 54 | PD>HC | - | - | - | 0.839 | <10^-3^ |
| MFG.R | 1080 | 45 15 48 | PD>HC | - | - | - | 0.832 | <10^-3^ |
| SFG.R | 1080 | 27 60 6 | PD>HC | - | -0.568 | - | 0.738 | 0.003 |

H-Y, Hoehn & Yahr Scale; AUC, area under the curve. CereAL, cerebellum anteriol lobe; PreCG, precentral gyrus; PoCG, postcentral gyrus; CUN, cuneus; PCUN, precuneus; CAL, calcarine fissure and surrounding cortex; SFG, superior frontal gyrus; MFG, middle frontal gyrus; ITG, inferior temporal gyrus; IPL, inferior parietal, but supramarginal and angular gyri; PCUN, precuneus; IFGtriang, inferior frontal gyrus, triangular part; SMA, supplementary motor area; INS, insula; CAU, caudate nucleus; ACG, anterior cingulated and paracingulate gyri; MCG, medial cingulated and paracingulate gyri; PCL, paracentral lobule. L, left; R, right. The superscripts indicate regions that are used to perform graph-based efficiency analyses and are visualized in Fig. 3A in the main text.
