# Supplementary material for: Widespread Increase of Functional Connectivity in Parkinson’s Disease with Tremor: A Resting-State fMRI Study
Source: Front Aging Neurosci. 2015 Feb 3;7:6. doi: 10.3389/fnagi.2015.00006 (PMC4315047; doi:10.3389/fnagi.2015.00006)
Supplement: Supplementary file 2 [file Table2.DOC]

**Table S2**. Summary of between-group differences of gray matter volume and their correlation with clinical performances and classification.

|  | **Between-group difference** | | | **Correlation** | | | | **Classification** | |
| --- | --- | --- | --- | --- | --- | --- | --- | --- | --- |
| Region | Vol (mm^3^) | Peak MNI  (x, y, z) | Direction | Duration | UPDRS | Tremor | H-Y | AUC | P |
| CC1/LING/  FFG.R | 48.13 | 15 -90 -24 | PD>HC | - | - | - | - | 0.876 | <10^-3^ |
| MOG.L | 63.75 | -28.5 -99 9 | PD<HC | - | - | - | - | 0.841 | <10^-3^ |
| INS/IFG.R | 123.75 | 45 6 9 | PD<HC | - | - | - | - | 0.820 | <10^-3^ |
| PHG/HIP/  AMYG.R | 102.88 | 24 -15 -13.5 | PD<HC | 0.631 | - | - | - | 0.807 | <10^-3^ |
| PHG/FFG.R | 53.25 | 36 -27 -16.5 | PD<HC | - | - | - | - | 0.795 | <10^-3^ |
| INS.L | 43.75 | -33 -1.5 7.5 | PD<HC | - | - | -0.605 | - | 0.784 | <10^-3^ |
| MTG/MOG.L | 57.75 | -58.5 -69 10.5 | PD<HC | - | - |  | - | 0.771 | <10^-3^ |
| MOG/PCUN.L | 37.13 | -28.5 -82.5 39 | PD<HC | - | -0.589 | - | -0.673 | 0.769 | <10^-3^ |
| MTG/STG.R | 58.25 | 61.5 -60 4.5 | PD<HC | - | - | - | - | 0.768 | <10^-3^ |
| ANG/SPL/  IPL/PCUN.R | 103.13 | 43.5 -72 48 | PD<HC | - | - | - | - | 0.759 | 10^-3^ |
| RG/IFG.R | 37.88 | 10.5 25.5 -28.5 | PD<HC | - | - | - | - | 0.717 | 10^-3^ |

Vol, cluster volume; x, y, z, coordinate of peak locations; UPDRS, Unified Parkinson’s Disease Rating Scale; H-Y, Hoehn & Yahr Scale; AUC, area under curve. PHG, parahippocampal gyrus; FFG, fusiform gyrus; RG, rectal gyrus; IFG, inferior frontal gyrus; CC1, cerebellum crus 1; LING, lingual gyrus; HIP, hippocampus; AMYG, amygdala; MTG, middle temporal gyrus; MOG, middle occipital gyrus; INS, insula; STG, superior temporal gyrus; ANG, angular gyrus; SPL, superior parietal lobule; IPL, inferior parietal lobule; PCUN, precuneus.
