# Supplementary figures and images for: Widespread Increase of Functional Connectivity in Parkinson’s Disease with Tremor: A Resting-State fMRI Study
Source: Front Aging Neurosci. 2015 Feb 3;7:6. doi: 10.3389/fnagi.2015.00006 (PMC4315047; doi:10.3389/fnagi.2015.00006)

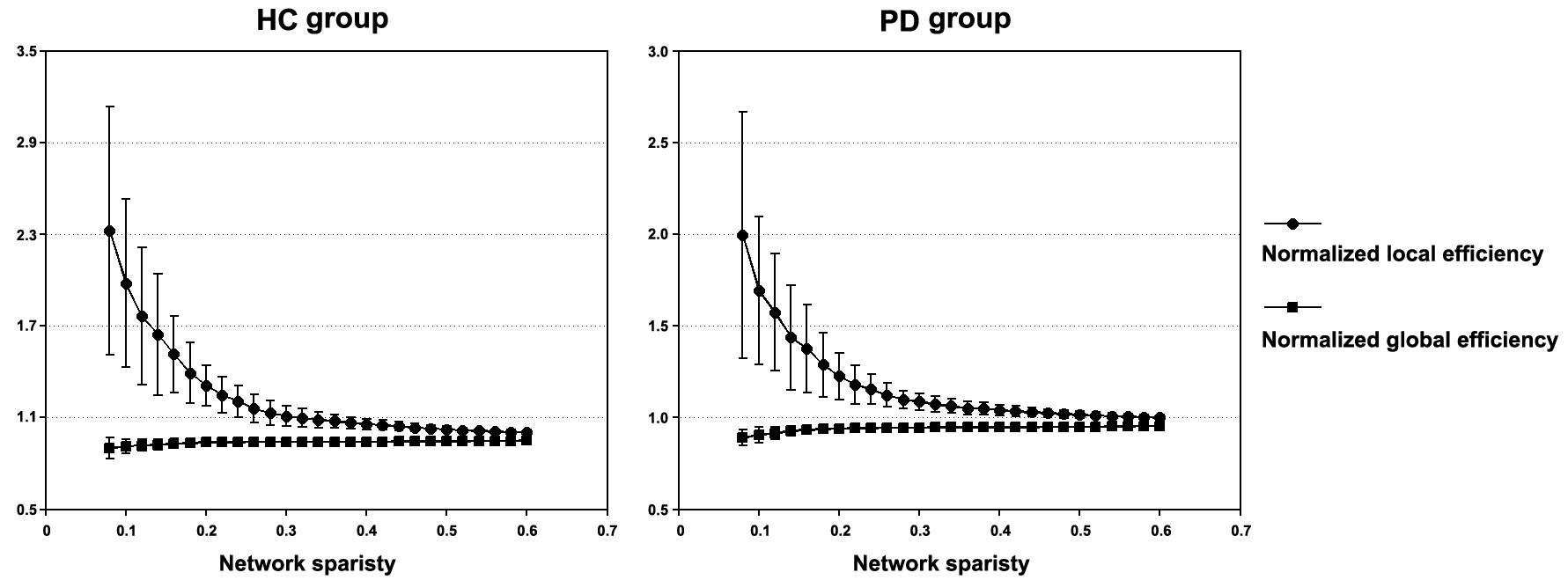

Supplement: Supplementary file 3 [file Image1.TIF]
